# Tolerance to drought and salt stress in plants: Unraveling the signaling networks

**DOI:** 10.3389/fpls.2014.00151

**Published:** 2014-04-22

**Authors:** Dortje Golldack, Chao Li, Harikrishnan Mohan, Nina Probst

**Affiliations:** Department of Biochemistry and Physiology of Plants, Faculty of Biology, Bielefeld UniversityBielefeld, Germany

**Keywords:** transcription factor, *Arabidopsis*, lipid signaling, ROS, drought, MAP kinase

## Abstract

Tolerance of plants to abiotic stressors such as drought and salinity is triggered by complex multicomponent signaling pathways to restore cellular homeostasis and promote survival. Major plant transcription factor families such as bZIP, NAC, AP2/ERF, and MYB orchestrate regulatory networks underlying abiotic stress tolerance. Sucrose non-fermenting 1-related protein kinase 2 and mitogen-activated protein kinase pathways contribute to initiation of stress adaptive downstream responses and promote plant growth and development. As a convergent point of multiple abiotic cues, cellular effects of environmental stresses are not only imbalances of ionic and osmotic homeostasis but also impaired photosynthesis, cellular energy depletion, and redox imbalances. Recent evidence of regulatory systems that link sensing and signaling of environmental conditions and the intracellular redox status have shed light on interfaces of stress and energy signaling. ROS (reactive oxygen species) cause severe cellular damage by peroxidation and de-esterification of membrane-lipids, however, current models also define a pivotal signaling function of ROS in triggering tolerance against stress. Recent research advances suggest and support a regulatory role of ROS in the cross talks of stress triggered hormonal signaling such as the abscisic acid pathway and endogenously induced redox and metabolite signals. Here, we discuss and review the versatile molecular convergence in the abiotic stress responsive signaling networks in the context of ROS and lipid-derived signals and the specific role of stomatal signaling.

## INTRODUCTION

Survival of plants under adverse environmental conditions relies on integration of stress adaptive metabolic and structural changes into endogenous developmental programs. Abiotic environmental factors such as drought and salinity are significant plant stressors with major impact on plant development and productivity thus causing serious agricultural yield losses (Flowers, 2004; Godfray et al., 2010; Tester and Langridge, 2010; Agarwal et al., 2013). The complex regulatory processes of plant drought and salt adaptation involve control of water flux and cellular osmotic adjustment via biosynthesis of osmoprotectants (Hasegawa et al., 2000; Flowers, 2004; Munns, 2005; Ashraf and Akram, 2009; Agarwal et al., 2013). Salinity induced imbalance of cellular ion homeostasis is coped with regulated ion influx and efflux at the plasma membrane and vacuolar ion sequestration (Hasegawa et al., 2000). Significantly, drought and salinity have additionally major detrimental impacts on the cellular energy supply and redox homeostasis that are balanced by global re-programming of plant primary metabolism and altered cellular architecture (Chen et al., 2005; Baena-González et al., 2007; Jaspers and Kangasjärvi, 2010; Miller et al., 2010; Zhu et al., 2010). In this review we focus on recent advances in understanding cellular signaling networks of biotechnological relevance in plant drought and salt adaptation. Here, we focus on induced rather than intrinsic tolerance mechanisms and do not explicitly distinguish between stress survival and tolerance. Known research findings on hormonal signal perception and transduction were integrated in the context of plant signaling networks under drought and salinity. We particularly aimed on reviewing links of drought and salt induced signal transduction to plant hormonal pathways, metabolism, energy supply and developmental processes.

## PLANT HORMONES: PIVOTAL ROLES IN PLANT STRESS SIGNALING

Plant hormones function as central integrators that link and re-program the complex developmental and stress adaptive signaling cascades. The phytohormone abscisic acid (ABA) functions as a key regulator in the activation of plant cellular adaptation to drought and salinity and has a pivotal function as a growth inhibitor (Cutler et al., 2010; Raghavendra et al., 2010; Weiner et al., 2010). Additionally, the view of function of ABA as a linking hub of environmental adaptation and primary metabolism is increasingly emerging. Intriguingly, ABA triggers both transcriptional reprogramming of cellular mechanisms of abiotic stress adaptation and transcriptional changes in carbohydrate and lipid metabolism indicating function of ABA at the interface of plant stress response and cellular primary metabolism (Seki et al., 2002; Li et al., 2006; Hey et al., 2010).

Abscisic acid signals are perceived by different cellular receptors and a concept of activation of specific cellular ABA responses by perception in the distinct cellular compartments is currently emerging. The nucleocytoplasmic receptors PYR/PYL/RCARs (PYRABACTIN RESISTANCE/ PYRABACTIN RESISTANCE-LIKE/REGULATORY COMPONENT OF ABA RECEPTORS) bind ABA and inhibit type 2C protein phosphatases (PP2Cs) such as ABI1 and ABI2 (Ma et al., 2009; Park et al., 2009). Inactivation of PP2Cs activates accumulation of active SNF1-RELATED PROTEIN KINASES (SnRK2s; Ma et al., 2009; Park et al., 2009; Umezawa et al., 2009; Vlad et al., 2009). The SnRK2s regulate ABA-responsive transcription factors including AREB/ABFs [ABA-RESPONSIVE PROMOTER ELEMENTS (ABREs) BINDING FACTORS (ABFs)] and activate ABA-responsive genes and ABA-responsive physiological processes (Umezawa et al., 2009; Vlad et al., 2009). Recently, function of plasma membrane-localized G protein-coupled receptor-type G proteins (GTGs) as ABA receptor in *Arabidopsis* has been shown (Pandey et al., 2009). Binding of ABA by GTG1/GTG2 and ABA hyposensitivity of GTG1/GTG2 *Arabidopsis* loss of function mutants supported a function of GTG1 and GTG2 as membrane-localized ABA receptors (Pandey et al., 2009). Extending the concept of involvement of GTG1 and GTG2 in ABA signaling, a role of the proteins in growth and development of *Arabidopsis* seedlings and in pollen tube growth by function as voltage-dependent anion channels has been reported (Jaffé et al., 2012). Thus, linking and dynamic integration of GTG1 and GTG2 in cellular ABA signaling and developmental regulation seems likely. Intriguingly, evidence for a third pathway of ABA perception has been emerging with the H subunit of Mg-chelatase (CHLH/ABAR). Integration of CHLH/ABAR in the cellular ABA signaling cascade as a chloroplastic ABA receptor and by plastid-to-nucleus retrograde signaling via the ABA responsive nucleocytoplasmic transcription repressor WRKY40 has been reported (Shen et al., 2006; Shang et al., 2010; Du et al., 2012). These findings strongly suggest contribution of a chloroplast-localized pathway to modulate cellular ABA signaling (Shen et al., 2006; Shang et al., 2010; Du et al., 2012).

Currently, increasing evidence has been emerging for modulation of ABA-mediated environmental signaling by interaction and competition with hormonal key regulators of plant cellular developmental and metabolic signaling. The complex and divergent endogenous and exogenous signals perceived by plant cells during development and environmental adversity are linked and integrated by distinct and interactive hormonal pathways. Particularly, convergence and functional modulation of ABA signaling by the plant growth regulating phytohormones gibberellic acid (GA) has a key regulatory function in the plant cellular network of stress and developmental signaling (Golldack et al., 2013). According to accepted concepts, in *Arabidopsis* GA signaling is mediated by binding of GA to GID1a/b/c that are GA receptor orthologs of the rice GA receptor gene *OsGID1* (*GA INSENSITIVE DWARF 1*; Ueguchi-Tanaka et al., 2005; Griffiths et al., 2006; Feng et al., 2008). GA responsive GRAS [for GA Insensitive (GAI), REPRESSOR of *ga1-3* (RGA), SCARECROW (SCR)] transcription factors function as major regulators in plant GA-controlled development. Cellular accumulation of the GRAS protein subgroup of DELLA proteins (GAI, RGA, RGL1, RGL2, RGL3) represses GA signaling and restrains growth and development (Cheng et al., 2004; Tyler et al., 2004; Yu et al., 2004). Interaction of DELLA proteins with the GA receptor GID1 induces degradation of the DELLA proteins and activates the function of GA (Cheng et al., 2004; Tyler et al., 2004; Yu et al., 2004). GA signals mediate binding of DELLA proteins to GID1 that is followed by conformational conversion of DELLA proteins. The modified DELLAs are recognized by the the F-box protein SLEEPY1 (SLY1) in *Arabidopsis* (Silverstone et al., 2001, 2007; Fu et al., 2002; Sasaki et al., 2003; Dill et al., 2004). Subsequently, DELLAs are polyubiquitinated by the SCFSLY1/GID2 ubiquitin E3 ligase complex and degraded via the 26S proteasome pathway (Silverstone et al., 2001; Fu et al., 2002; Sasaki et al., 2003; Dill et al., 2004).

A linking function of DELLA proteins at the interface of ABA-mediated abiotic stress responses and GA-controlled developmental signaling has been supported by modified salt tolerance of the quadruple DELLA mutant with functional losses of *rga, gai, rgl1*, and *rgl2* (Achard et al., 2006). Interestingly, the RING-H2 zinc finger factor *XERICO* regulates tolerance to drought and ABA biosynthesis in *Arabidopsis* (Ko et al., 2006). In addition, XERICO is a transcriptional downstream target of DELLA proteins indicating function of XERICO as a node of plant abiotic stress responses and development by linking GA and ABA signaling pathways (Ko et al., 2006; Zentella et al., 2007; Ariizumi et al., 2013).

**FIGURE 1:**
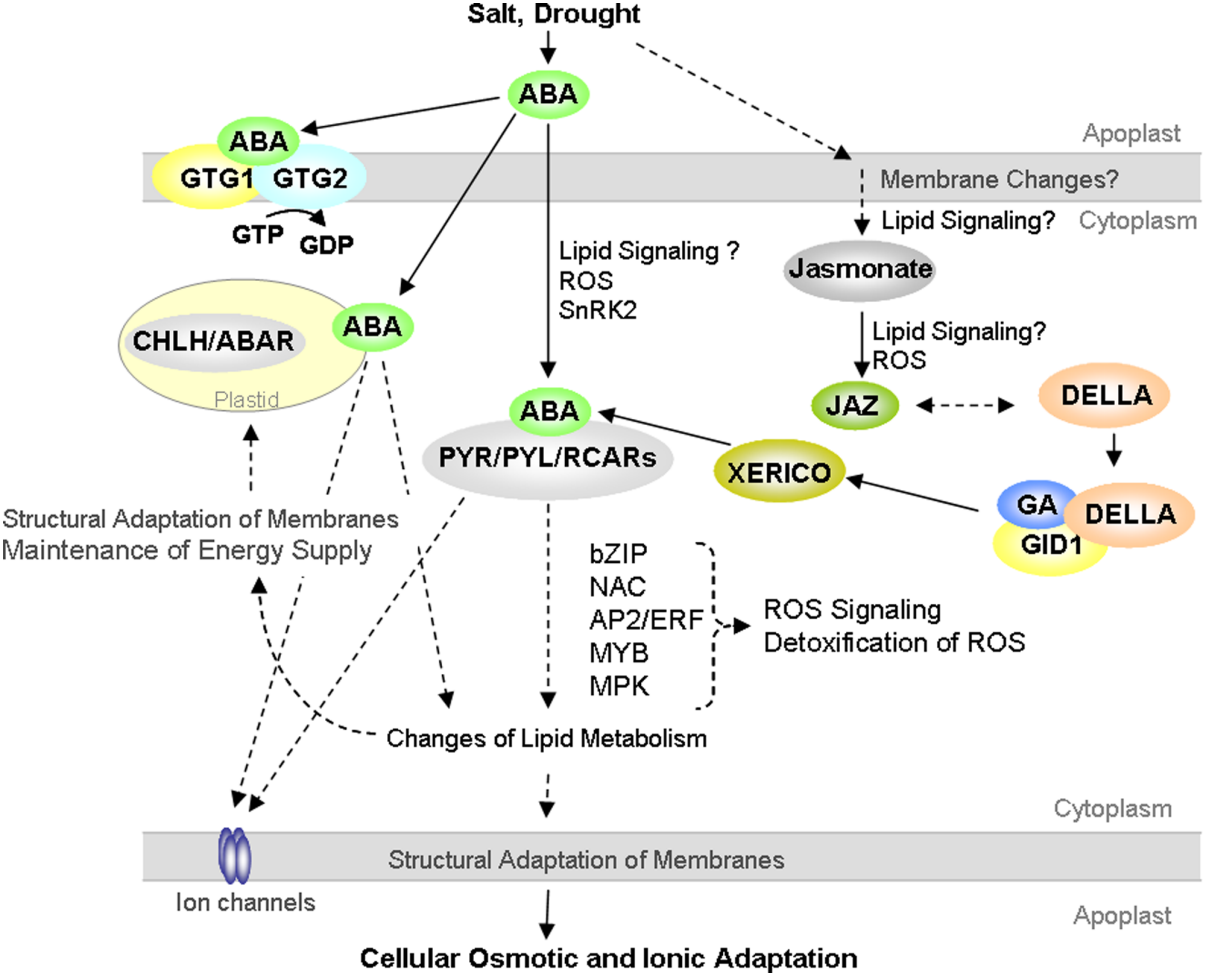
**Proposed model on crosstalk of abscisic acid (ABA), gibberellic acid (GA), and jasmonate signaling in plant cellular responses to the abiotic stressors drought and salt.** Hypothesized links are illustrated with dashed lines. The lines and arrows illustrate pathways that are not shown and described in detail. Compare text for details.

Recently, interesting evidence has been also provided for a convergence and crosstalk of GA and ABA signaling with the developmental regulator jasmonate in plant responses to drought. Jasmonates are membrane-lipid derived metabolites that originate from linolenic acid and have signaling functions in plant growth and biotic stress responses (e.g., Wasternack, 2007; Wasternack and Hause, 2013). Drought-induced transcriptional regulation of the rice JA receptor protein *OsCOI1a* (CORONATINE INSENSITIVE 1) and of key regulators of JA signaling *OsJAZ* (jasmonic acid ZIM-domain proteins) indicate significant integration of JA metabolism and signaling in plant abiotic stress responses (Du et al., 2013a; Lee et al., 2013). Importantly, expression of the DELLA protein RGL3 responds to JA, and additionally RGL3 interacts with JAZ proteins (Wild et al., 2012). These recent research advances emphasize function of DELLAs as an interface of ABA, GA and jasmonic acid signaling and suggest pivotal functional involvement of lipid-derived signaling in abiotic stress responses (**Figure 1**).

## MAJOR PLANT TRANSCRIPTION FACTOR FAMILIES: KEY PLAYERS IN THE REGULATORY NETWORKS UNDERLYING PLANT RESPONSES TO ABIOTIC STRESS

Comprehensive research on diverse abiotic stress responsive transcription factors shed light on the cellular mechanisms defining plant environmental adaptation (Golldack et al., 2011). Significantly, the majority of ABA-regulated genes share the conserved ABA-responsive *cis* element (ABRE; Yamaguchi-Shinozaki and Shinozaki, 2005, 2006). Besides the AREB/ABF (ABA-responsive element binding protein/ABRE-binding factor) family, the DREB/CBF subfamily of the AP2/ERF transcription factors has a central function in regulating plant adaptation to adversity via ABA dependent and independent pathways (Yamaguchi-Shinozaki and Shinozaki, 2005, 2006). Significant evidence for a linking function of DREB/CBF in integrating environmentally derived signals and plant development was early provided by DREB/CBF overexpressing *Arabidopsis* with increased tolerance to drought, salt, and cold that was counterbalanced by serious developmental defects (Kasuga et al., 1999). Supporting this functional connection, cold responsive CBF1 regulated GA biosynthesis and accumulation of the DELLA protein RGA thus suggesting integration of AP2/ERF in abiotic stress signaling and GA-regulated plant development (Achard et al., 2008). The bZIP-type AREB/ABF transcription factors AREB1, AREB2, and AREB3 target cooperatively ABRE-dependent gene expression via a suggested interaction with the sucrose non-fermenting 1-related protein kinase 2 (SnRK2) protein kinase SRK2D/SnRK2.2 (Yoshida et al., 2010). In addition, the *Arabidopsis* transcription factor *bZIP24* controls reprogramming of a broad array of salinity dependent and developmental gene expression indicating a pivotal role of the factor in maintaining plant development under conditions of adversity (Yang et al., 2009).

The view of an integrative function of many transcription factors in linking and balancing related or seemingly unrelated cellular responses is further supported by other drought and salt responsive transcription factors. Intriguingly, the picture is increasingly emerging that plant signaling does not function as independent and paralleled pathways but cellular crosstalks and hubs within the signaling network exist. The view is increasingly emerging that stress adaptive signaling is tightly linked to the cellular primary metabolism, energy supply and developmental processes. Thus, the tomato *NAC*-type (*NAM, ATAF1,2, CUC2*) transcription factor *SlNAC1* was responsive to multiple abiotic and biotic stresses (Ma et al., 2013). Regulation of the factor by ABA, methyl jasmonate, gibberellin, and ethylene indicates a node role of the factor in diverse signal transduction pathways in tomato (Ma et al., 2013). The ABA-responsive NAC-transcription factor *VNI2* (*VND-INTERACTING1)* is a repressor of xylem vessel formation and has additional functions in leaf aging thus integrating plant senescence to ABA signaling (Yang et al., 2011). As another example, the *NAC* transcription factor *ANAC042* (*JUB1, JUNGBRUNNEN 1*) links leaf senescence to hyperosmotic salinity response and is involved in H_2_O_2_ signaling (Wu et al., 2012). Over-expression of the drought and ABA responsive rice NAC-type transcription factor *OsNAC10* allowed identification of NAC dependent target genes that included AP2 and WRKY-type transcription factors (Jeong et al., 2010). These findings strongly indicate a hub role of NAC transcription factors in stress relevant hierarchic regulatory pathways.

Drought and ABA-responsive NAC factors are likely to control and link subclusters of cellular stress adaptation processes under control of diverse subsets of specific transcription factors such as members of the AP2 and WRKY families. Thus, hypersensitivity to drought of an *Arabidopsis WRKY63* loss of function mutant was related to reduced ABA sensitivity in guard cells indicating specific control of abiotic stress adaptation by this WRKY transcription factor (Ren et al., 2010). ABA and salt responsive *Arabidopsis WRKY33* downstream targets genes with functions in detoxification of reactive oxygen species (ROS) such as glutathione *S*-transferase *GSTU11*, peroxidases, and lipoxygenase *LOX1* (Jiang and Deyholos, 2009). According to the involvement of WRKY33 in osmotic stress responses, ROS detoxification and ROS scavenging, a role of WRKY controlled cellular ROS levels in abiotic stress signaling seems likely. Extending and supplementing this concept, the WRKY-type transcription factor *ThWRKY4* from *Tamarix hispida* controls cellular accumulation of ROS via regulating expression and activity of antioxidant genes such as superoxide dismutase and peroxidase (Zheng et al., 2013). Modified tolerance of *ThWRKY4* overexpressing plants to salt and oxidative stress was referred to *ThWRKY4*-mediated cellular protection against toxic ROS levels (Zheng et al., 2013). Accordingly, an involvement of WRKY in linking osmotic and oxidative stress defense as well as in ROS mediated signaling crosstalks is suggested.

Another crucial and undervalued mechanism of plant adaptation to drought and salinity is the maintenance of cell wall development and generation of the extracellular matrix in terms of plant development and of protection against water loss. Intriguingly, transcriptional expression of the *Arabidopsis* R2R3-MYB transcription factor *AtMYB41* was induced by drought, salt, and ABA (Cominelli et al., 2008; Lippold et al., 2009). Modified drought sensitivity of *AtMYB41* overexpressing *Arabidopsis* was linked to lipid metabolism, cell wall expansion, and cuticle deposition demonstrating a key function of *AtMYB41* in plant drought protection and survival via primary lipid metabolism and cuticle formation (Cominelli et al., 2008). Recently, function of *AtMYB41* was also linked to primary carbon metabolism indicating a relationship between cuticle deposition, plant tolerance against desiccation as well as cellular lipid and carbon metabolism (Cominelli et al., 2008; Lippold et al., 2009). The salt-responsive rice R2R3-type MYB transcription factor *OsMPS* (*MULTIPASS*) targets genes with function in biosynthesis of phytohormones and of the cell-wall (Schmidt et al., 2013a). These recent research advances highlight the importance of a functional plant extracellular matrix and of cuticular polymer biosynthesis for plant salt and drought adaptation. Accordingly, a key function of stress responsive transcription factors in integrating cuticle formation in the cellular primary metabolism in response to environmental adversity is supported and likely.

## LIPIDS: STILL AN ENIGMA IN ABIOTIC STRESS ADAPTATION AND STRESS DERIVED SIGNALING?

Plant adaptation to a changing water and ionic status in the surrounding environment requires rapid and sensitive sensing of the stress situation and stress induced signaling. A crucial and existential challenge for plant cells is the maintenance of integrity of cellular membranes both at the plasma membrane and of the enodomembranes. Thus, plants ensure homeostasis of metabolism and cellular energy supply. Additionally, increasing evidence for pivotal involvement of lipid-derived signaling in primary sensing of environmental changes and in triggering and regulating cellular hormonal signaling cascades has been emerging (**Figure 1**). Interestingly, vice versa ABA transcriptionally downstream targets lipid metabolism and lipid transfer proteins suggesting tight interaction of ABA-dependent signaling and lipid metabolic pathways to maintain structure and function of cellular membranes (Seki et al., 2002; Li et al., 2006). Thus, ABA-triggered modification of primary lipid metabolism contributes unequivocally to stress adaptive reorganization of membranes and to the maintenance of cellular energy supply under abiotic stress conditions and limitation in water supply. Increased transpirational water loss of *Arabidopsis* mutants with a functional knock out of *LTP3* (*Lipid Transfer Protein 3*) suggests lipid-based adaptive changes of membranes and the plant cuticle to regulate water loss and transpiration under drought (Guo et al., 2013).

Drought-induced changes of monogalactosyldiacylglycerol (MGDG) and digalactosyldiacylglycerol (DGDG) contents in the chloroplast envelope and in thylakoid membranes in cowpea *(Vigna unguiculata*) have been suggested to stabilize and maintain lamellar bilayer structure and thus the function of chloroplasts under drought stress (Torres-Franklin et al., 2007). In support of these findings, changes of MGDG in the drought tolerant resurrection plant *Craterostigma plantagineum* during desiccation are likely to contribute to membrane stabilization and to the maintenance of photosynthetic energy supply (Gasulla et al., 2013). The *Arabidopsis* cold-responsive *SFR2* (*SENSITIVE TO FREEZING 2*) mediates removal of monogalactolipids from the chloroplast envelope membrane and stabilizes membranes during freezing indicating that structural re-shaping of chloroplast membranes is an essential and general mechanism of plant cellular dehydration responses (Moellering et al., 2010).

Next to strong evidences for a fundamental importance of lipid mediated re-organization of cellular membranes to cope with changes in the plant water status, also comprehensive evidence for functions of lipid signaling in plant drought and salt responses has been emerging. In rice, levels of PIP2 (phosphatidylinositol bisphosphate), PA (phosphatidic acid), and DGPP (diacylglycerolpyrophosphate) increased upon salt stress (Darwish et al., 2009). Based on these findings involvement of phospholipase C and diacylglycerol kinase in salt stress induced signaling has been hypothesized (Darwish et al., 2009). Function of phospholipase C was linked to ABA signaling and stomatal regulation indicating a functional role of phosphoinositides in guard cell signaling (Hunt et al., 2003; Mills et al., 2004). The inositol phosphate myo-inositol hexakisphosphate (InsP6) has a role as an ABA-responsive signaling molecule that regulates stomatal closure via cellular calcium and the plasma membrane potassium conductance (Lemtiri-Chlieh et al., 2003). Phosphoinositides have key roles in regulating membrane peripheral signaling proteins and influence the activity of integral proteins and ion channels (Suh et al., 2006; Falkenburger et al., 2010). Importantly, work on inhibitors of phosphoinositide-dependent phospholipases C (PI-PLCs) in *Arabidopsis* has provided considerable insight in the drought stress related lipid signaling by identifying links of phosphoinositides to the DREB2 pathway (Djafi et al., 2013).

A role of lipid-derived messengers in ABA signaling was also evident by ACBP1 (acyl-CoA-binding protein 1) regulated expression of PHOSPHOLIPASE Dα1 (PLDα1; Du et al., 2013b). PHOSPHOLIPASE Dα1 has a function in the biosynthesis of the ABA regulating lipid messenger PA indicating that modulation of cellular lipid profiles is essential for regulation of abiotic stress related ABA signaling (Du et al., 2013b; Jia et al., 2013; Lu et al., 2013).

## SnRK2 AND MAPK: ANOTHER CHAPTER IN PLANT ABIOTIC STRESS SIGNALING

Protein kinases of diverse types and families are central integrators of plant abiotic stress signaling that link cellular metabolic signaling to stress adaptive physiological processes as regulation of ionic and osmotic homeostasis and to concerted changes of ROS in stressed plant cells (**Figure 1**). Accepted models emphasize hub functions of yeast sucrose non-fermenting 1 (SNF1) serine-threonine protein kinase, homologous mammalian AMP-activated protein kinase (AMPK) and plant SnRKs [Snf (sucrose non-fermenting)-1-related protein kinases] in the cellular carbon and energy metabolism (Halford and Hey, 2009). In plants, SnRK1 subgroup kinases have reported functions in metabolic signaling and development (Zhang et al., 2001; Halford et al., 2003). Considerable insight into protein kinase functions in plant abiotic stress adaptation has been provided by elucidation of the SOS pathway with central functions in maintenance and regulation of ion homeostasis under salt stress. Intriguingly, the SnRK3 SOS2-like (Salt Overly Sensitive3) protein kinases interact with SOS3-like calcium-binding proteins to activate the plasma membrane Na^+^/H^+^ antiporter SOS1 via the SOS pathway (Chinnusamy et al., 2004; Du et al., 2011). Recent research highlights direct interaction of SnRK2.8 and the ABA responsive NAC (NAM/ATAF1/2/CUC2) transcription factor NTL6 indicating integration of a SnRK2-type kinase in the ABA controlled cellular framework of abiotic stress adaptation (Kim et al., 2012). Extending these findings, in rice, the SnRK2 kinase *SAPK4* links regulation of ion homeostasis to scavenging of ROS thus suggesting interaction of ionic and oxidative stress signaling pathways in plant adaptation to adversity (Diédhiou et al., 2008). Consistent with these findings, a node function of SnRK2-type kinases in ABA signaling and ROS generation has been elucidated in stomatal guard cells. The ABA responsive SnRK2 *OST1* (*OPEN STOMATA 1*) regulates stomatal closure by modulating the cellular production of H_2_O_2_ via NADPH oxidases (Sirichandra et al., 2009; Vlad et al., 2009). *Arabidopsis* OST1 mutants provided evidence for a role of *OST1* in the regulation of inward K^+^ channels, Ca^2^^+^ -permeable channels and the slow anion channel *SLAC1* thus supporting a hub function of *OST1* in linking ABA, ion channels and NADPH oxidases in the regulation of stomatal apertures in guard cells (Sirichandra et al., 2009; Vlad et al., 2009; Acharya et al., 2013). As a fascinating finding, the *Arabidopsis snrk2.2/2.3/2.6* triple-mutant with decreased sensitivity to ABA allowed identification of SnRK2 phosphorylation targets that included proteins with functions in chloroplasts, in signal transduction and in the regulation of flowering (Wang et al., 2013). These research advances provide insights in SnRK2-mediated regulatory crosstalks and interactions of developmental, metabolic and stress adaptive processes in the plant cellular signaling framework.

Recent advances on mitogen-activated protein kinase (MAPK) mediated signal transduction cascades have provided another pivotal understanding of the integration of physiological and cellular responses to environmental adversity. MAPK cascades functionally link MAP3Ks (MAP2K kinase) serine/threonine kinases, MAP2K (MAPK kinase) dual-specificity kinases and MAPK serine/threonine kinases (Colcombet and Hirt, 2008). As an accepted concept of functional importance in abiotic stress adaptation, involvement of MAPKs in drought and salt adaptation have been reported for wide ranging plant species such as rice, *Arabidopsis* to alfalfa *SIMK* and *SIMKK* (Kiegerl et al., 2000; Ning et al., 2010; Yu et al., 2010). Recent research highlights a central role of *Arabidopsis* MKK4 in the osmotic stress response by regulation of MPK3 activity, accumulation of ROS and targeting the ABA biosynthetic process via *NCED3* (*NINE-CIS-EPOXYCAROTENOID DIOXYGENASE 3*; Kim et al., 2011). Several studies indicated a hub function of MPK6 as another member of the MAPK cascade in linking of osmotic stress responses to ROS and oxidative bursts. Thus, recent research has identified abiotic stress induced ROS accumulation under control of MPK6, MKK1, and MKKK20 supporting a dynamic control of the signaling component ROS by MPK6 and other components of the MAPK pathway (Xing et al., 2008; Kim et al., 2012).

Novel findings uncover links of the MAPK cascade to cellular lipid transfer processes indicating a coupling of MAP-type kinases to stress adaptive changes of membranes, intracellular membrane trafficking or probably to stress-dependent lipid signaling. Thus, recent research advances proved direct regulation of MPK6 mediated phosphorylation of the plasma membrane Na^+^/H^+^ antiporter *SOS1* by NaCl and by PA supporting relationships of lipids to MAPK signaling in plant salt stress responses (Yu et al., 2010). Integration of MPK6 in differential signaling pathways has been additionally reported by interaction of MPK6 with the *Arabidopsis* C2H2-type zinc finger protein *ZAT6* that functions both in plant developmental processes and in osmotic stress responses (Liu et al., 2013b). In several recent studies, emphasis has been placed on detailed characterization of co-regulation and interaction of the MAP kinase pathway and ROS signaling within the cellular signaling framework thus further strengthening the understanding of MAP kinase as a hub in signaling under environmental adversity. In rice, the salt responsive MAPK cascade is linked to ROS signaling by the transcription factor *SERF1* (*salt-responsive ERF1*; Schmidt et al., 2013b). Cotton MAPK *GhMPK16* is functionally involved in pathogen resistance, drought tolerance and ROS accumulation indicating a role of *GhMPK16* as an interface between biotic and abiotic stress signaling (Shi et al., 2011).

## ROS SIGNALING IN PLANTS UNDER DROUGHT AND SALT STRESS

Current concepts emphasize a central function of cellular ROS as a signaling interface in plant drought and salt adaptation hat links stress signals to regulation of metabolism and the cellular energy balance (**Figure 1**). Significantly, environmental adversity such as drought and salinity impairs cellular ionic and osmotic homeostasis but additionally compromises photosynthesis, cellular energy depletion, and redox imbalances (e.g., Baena-González et al., 2007; Abogadallah, 2010; Jaspers and Kangasjärvi, 2010; Miller et al., 2010; Zhu et al., 2010). Excess generation and accumulation of ROS such as superoxide, hydrogen peroxide and nitric oxide cause oxidative damages in the apoplastic compartment and damages of cellular membranes by lipid peroxidation and have an extensive impact on ion homeostasis by interfering ion fluxes (Baier et al., 2005). Excess ROS amounts are particularly scavenged by antioxidant metabolites such as ascorbate, glutathione, tocopherols and by ROS detoxifying enzymes as superoxide dismutase, ascorbate peroxidase, and catalase (Mittler, 2002; Neill et al., 2002). Current models emphasize a dual regulatory function of ROS as a signaling molecule in plant drought and osmotic stress tolerance by sensing the cellular redox state and in retrograde signaling. Studies on transcription factors of the WRKY and basic-helix-loop helix types enhanced the understanding of crosstalks of osmotic and oxidative stress responsive signaling pathways significantly. Thus, *Arabidopsis WRKY33* responds to osmotic and oxidative stresses (Miller et al., 2008). Regulatory function of *bHLH92* and *WRKY33* in ROS detoxification by targeting peroxidases and glutathione-*S*-transferases suggested a function of the transcription factors in linking ROS scavenging to osmotic and oxidative stress induced signaling (Miller et al., 2008; Jiang and Deyholos, 2009; Jiang et al., 2009). Recent research advances linked the regulation of *Arabidopsis* salt and osmotic stress tolerance to ROS-responsive *WRKY15* and mitochondrial retrograde signaling (Vanderauwera et al., 2012). Another recent advance in understanding the importance of ROS in plant salt responses was the discovery of a coupled function of plastid heme oxygenases and ROS production in salt acclimation (Xie et al., 2011). These findings strongly suggest involvement of the chloroplast to nucleus signaling pathway in plant salt adaptation (Xie et al., 2011). Additionally, work on cross-species expression of a SUMO conjugating enzyme has provided considerable insight into the links of ROS, ABA dependent signaling and the sumoylation pathway in plant salt and drought tolerance (Karan and Subudhi, 2012). Functional relation of the maize bZIP transcription factor *ABP9*, glutamate carboxypeptidase *AMP1*, and the ankyrin-repeat protein ITN1 to ABA signaling, ROS generation and ROS scavenging further support interaction and correlation of ABA and ROS related pathways as signaling nodes in plant adaptation to drought and salt (Sakamoto et al., 2008; Zhang et al., 2011; Shi et al., 2013).

## THE SPECIFIC FUNCTION OF STOMATAL SIGNALING IN PLANT DROUGHT AND SALT TOLERANCE

Constant dynamic regulation of stomatal aperture is obligatory for successful adaptation of plants to abiotic stresses. Prevention of excess water loss via transpiration depends on reliable adjustment of stomatal closure to environmental adversity. Hence, elucidation of sensing and signaling in stomatal guard cells has been attracting particular attention to understand regulation of stomatal conductance under conditions of drought and salinity. As another example, in maize mutants of the E3 ubiquitin ligase *ZmRFP1*, enhanced drought tolerance and decreased ROS accumulation indicated linked regulation of stomatal closure and ROS scavenging (Liu et al., 2013a). The *Arabidopsis* plasma membrane receptor kinase, *GHR1* (*GUARD CELL HYDROGEN PEROXIDE-RESISTANT1*) linked ABA and H_2_O_2_ signaling in stomatal closure (Hua et al., 2012). In addition, *GHR1* regulated an S-type anion channel suggesting a node function of this receptor kinase in ion homeostasis, ABA and H_2_O_2_ mediated signaling pathways in guard cells (Hua et al., 2012).

As aforementioned, the SnRK2 protein kinase *OST1* (*SnRK2 OPEN STOMATA 1*) is a central regulator of stomatal aperture and links guard cell movement to the ABA signaling network (Sirichandra et al., 2009). *OST1* targets NADPH oxidases, inward K^+^ channels, Ca^2^^+^ -permeable channels and the slow anion channel *SLAC1* in stomatal guard cells (Sirichandra et al., 2009; Vlad et al., 2009; Acharya et al., 2013). In addition, the SnRK2 protein kinase *OST1* also targets voltage-dependent quickly activating anion channels of the R-/QUAC-type in guard cells (Imes et al., 2013). These data suggest coordinated control of SLAC1-mediated transport of chloride and nitrate and QUAC1-mediated transport of malate in the same ABA signaling pathway (Imes et al., 2013). Recently, the finding of direct dephosphorylation of SLAC1 by the PP2C (protein phosphatase 2C) *ABI1* provided interesting evidence for a specific alternative regulatory mechanism of the anion channel SLAC1 (Brandt et al., 2012).

Recent research uncovered co-regulation of ABA-induced stomatal closure, guard cell H^+^-ATPase and Mg-chelatase H subunit (*CHLH*; Tsuzuki et al., 2013). CHLH/ABAR is involved in the chlorophyll biosynthetic process and a function of CHLH/ABAR as a chloroplastic ABA receptor via plastid-to-nucleus retrograde ABA signaling has been suggested (Shen et al., 2006; Shang et al., 2010; Du et al., 2012). In *Arabidopsis*, functional mutation of CHLH affected phosphorylation of H^+^-ATPase and blue light dependent stomatal regulation (Tsuzuki et al., 2013). These findings validate importance of *CHLH* in linking the ABA signaling network to the regulation of ionic homeostasis and blue light responses in guard cells and plant drought tolerance (Tsuzuki et al., 2013). Interestingly, ABA-dependent regulation of stomatal closure responds to mutation of the phosphate transporter *PHO1* and the vacuolar H^+^-ATPase subunit A (Zimmerli et al., 2012; Zhang et al., 2013). Again, these results support interaction and co-regulation of ion homeostasis in guard cells via ion transport, ABA signaling, and regulation of stomatal aperture (Zimmerli et al., 2012; Zhang et al., 2013). Intriguingly, the transporter *ZIFL1* (*Induced Facilitator-Like 1*) mediates potassium fluxes and has a dual function in regulating both cellular auxin transport and stomatal closure (Remy et al., 2013).

In conclusion, recent research advances have elucidated a molecular cellular signaling network for the understanding how plants control and regulate adaptation to the abiotic stresses drought and salinity. Essentially, molecular signaling components in plant adaptation to environmental adversity have been connected to hub transcription factors, MAPK pathways, ROS and lipid-derived pathways. Importantly, it is expected that further and perspective advances in the network modeling of cellular abiotic stress signaling will provide new and efficient strategies for improving environmental tolerance in crops.

## Conflict of Interest Statement

The authors declare that the research was conducted in the absence of any commercial or financial relationships that could be construed as a potential conflict of interest.
